# Attachment and Detachment of Oil Droplets on Solid Surfaces: Insights from Molecular Simulations

**DOI:** 10.3390/ijms252111627

**Published:** 2024-10-29

**Authors:** Małgorzata Borówko, Tomasz Staszewski

**Affiliations:** Department of Theoretical Chemistry, Institute of Chemical Sciences, Faculty of Chemistry, Maria Curie-Skłodowska University in Lublin, 20-031 Lublin, Poland; staszewski@umcs.pl

**Keywords:** molecular dynamics, wettability, adsorption, Janus nanoparticles, enhanced oil recovery

## Abstract

The behavior of oil droplets at solid surfaces is a key aspect of oil production and environmental protection. In this paper, the mechanisms of attachment and detachment of oil aggregates are studied via molecular dynamics simulations. The influence of oil–surface interactions on the shape and structure of adsorbed clusters is discussed. Using selected shape metrics, we prove quantitatively that the shape of oil aggregates changes from almost spherical droplets, through multilayer structures, to monolayer films. The oil detachment from solid surfaces plays a major role in enhanced oil recovery. Here, we investigated oil droplet detachment from the solid surface immersed in Janus nanoparticle suspensions. The nanoparticle is modeled as a dimer built of segments that exhibit different affinities to oil and solvent molecules. Our results indicated that the adsorption of Janus dimers on the surface of oil droplets played an essential role in the oil removal processes. Stronger adsorption causes faster detachment of the oil droplet. Based on our findings, suspensions of Janus dimers can be considered to be high-performance agents in removing oil droplets from solid surfaces.

## 1. Introduction

The removal of oil droplets from solid surfaces is of great importance in many technological processes, such as enhancing oil recovery (EOR), the cleaning of metal and semiconductor surfaces, detergency, microfluidic devices, and so on. Among them, EOR is a hot topic in the oil industry and is attracting a lot of attention [1]. There are three stages of completion of crude oil extraction processes: primary, secondary, and tertiary oil recovery [2]. The primary oil recovery process is caused by the natural driving forces of the reservoir, namely rock and fluid expansion, solution gas, water influx, gas cap, and gravity drainage [3]. However, only 10% of oils out of total deposits can be recovered from the reservoir by means of these traditional extraction methods [4]. In the secondary recovery process, external fluids like water and immiscible gas are injected to recover more oil via a volumetric sweep of the original oil in place [5]. The primary and secondary oil recovery processes mainly target mobile oil in the reservoir. However, the tertiary recovery method targets immobile oil remaining within the reservoir after the secondary recovery process is completed. This stage of the process is usually called “enhanced oil recovery (EOR)” and is generally completed by injecting chemicals, gases, and thermal energy [6]. In EOR processes, injected fluids interact with the rock, oil, and brine system within the reservoir, altering the system properties and, in turn, oil production efficiency. The major EOR processes, main mechanisms, and challenges are described in detail in the review [1].

The chemical EOR methods are becoming very popular due to their technical and economic feasibility [1]. The addition of suitable chemical agents can cause wettability alteration, interfacial tension reduction, a change in the mobility ratio, and influences on many other parameters characterizing the system. A key task is to control the wettability of the reservoir surface. The wettability alteration is carried out to make the rock reservoir surface water-wet from oil-wet, leading to easy oil extraction. Several reviews have been published on different aspects of the wettability alteration [1,7,8,9]. This is mostly achieved by chemical flooding, which includes surfactant flooding, injection of polymer solutions, alkaline flooding, and nanofluid flooding.

In the last few decades, extensive research has been conducted to alter the wettability by chemical flooding, especially with different surfactants and nanofluids. These studies showed that surfactants exhibit satisfactory effectiveness in increasing oil recovery only under mild, low temperature, and low salinity, reservoir conditions. Moreover, the high costs associated with surfactant flooding make it difficult to use this method on a large scale [10,11]. To overcome the limitations associated with surfactant flooding, the application of nanoparticles has been proposed [1]. Nanoparticles, characterized by their nanometer-scale size and large surface area, have properties that make them well-suited for application in chemical EOR. In comparison to surfactant flooding, the utilization of modified nanoparticles can reduce the costs of oil recovery and environmental pollution.

Results from many nanoparticle flooding experiments have shown improved oil recovery using different types of nanoparticles [1]. However, relatively few studies have been conducted to understand its mechanism and the performance-affecting parameters for EOR. In the literature, various mechanisms such as wettability alteration, reduction in interfacial tension, increased viscosity of flooding solution, viscosity reduction in reservoir oil, and log jamming are found to be responsible for nanofluid-assisted EOR. Among them, the wettability alteration is considered to be the most important mechanism. Nanofluids effectively transform the oil-wet reservoir into more water-wet. The experimental studies showed that disjoining pressure is an underlying phenomenon for the alteration of wettability by nanofluids [12]. Disjoining pressure can be defined as the required pressure gradient to remove the fluid adhered to the reservoir surface due to the adhesion force between the fluid and the solid surface. During a disjoining process, nanoparticles in the fluid create a wedge film structure at the three-phase contact area that spreads further on the rock surface and changes its wettability. Another phenomenon that affects the wettability is the adsorption of nanoparticles at a rock surface [13,14]. Moreover, the effect of nanoparticles on interfacial tension (IFT) has been receiving attention from many researchers [15].

Although experimental studies can provide quantitative data on the effects of nanoparticles in enhancing oil recovery, they do not give a picture of the process at the microscopic level. To investigate the mechanism underlying these processes, molecular simulation can be a well-chosen tool. Recently, molecular simulations have been applied to study the systems involving surfactants and nanoparticles in the chemical EOR field [16,17,18,19,20]. The synergistic effects of surfactants and nanoparticles on oil–water interfacial behaviors were studied using molecular simulation [16]. Moreover, Vu and Papavassiliou [17] showed that Janus nanoparticles had the capability to reduce the IFT of the oil–water systems containing surfactants. Although the synergy between nanoparticles and surfactants offers the potential to reduce surfactant usage in EOR, the presence of surfactants still poses environmental concerns. A very promising class of solid-active agents are Janus particles that can be treated as nanosurfactants because their surfaces have both hydrophilic and hydrophobic patches [21]. Liang et al. [18] compared the oil displacement properties of nanoparticles with grafted hydrophobic, hydrophilic, and a combination of hydrophilic and hydrophobic chains. They showed that all considered nanoparticle fluids exhibited enhanced oil droplet detachment. The nanoparticles with mixed hydrophilic and hydrophobic chains had optimal EOR capability. Chang and his co-workers [19] studied the displacement mechanism of trapped oil in a rough channel by injecting different nanofluids. Their results proved that hydrophilic and Janus nanoparticles enhance the oil displacement efficiency by different mechanisms; Janus nanoparticles were capable of reducing the interfacial tension and altering the surface wettability. Zhou et al. [20] employed molecular dynamics simulations to explore the impact of modified ${\mathrm{SiO}}_{2}$ nanoparticles on oil–water interfacial behaviors and the detachment of oil droplets from an oil-wet surface. They analyzed the mechanism of oil detachment and discovered the formation of water channels that support this process. Moreover, their comparative analysis indicated that in terms of oil displacement efficiency, the thickness of the interfacial layer has a more significant impact than interfacial tension reduction.

Despite intensive research on oil recovery, the mechanism of this process is still unclear. The basic issue is the adsorption of oil on solid surfaces and the behavior of attached droplets or layers under various conditions. In this work, we present the results of systematic research on the formation of droplets on various surfaces. We employed molecular dynamics simulations which are a powerful tool for the molecular-scale understanding of the wettability of solids. The behavior of oil aggregates on surfaces is discussed in detail. Moreover, we studied the detachment of oil droplets from the surface caused by the addition of Janus nanoparticles. The effect of the interactions of Janus dimers [22,23,24] with the solvent on the performance of oil detachment is discussed. This study may provide some insight into the design of Janus nanoparticles with the desired properties for EOR applications and environmental pollution improvement.

## 2. Results and Discussion

### 2.1. Description of the Systems Studied

We consider two types of model systems, oil droplets adsorbed on solid surfaces from pure solvents and the solvents containing suspended Janus nanoparticles. In all systems, the density of polymer molecules (oil) is very low in comparison to the density of solvent molecules (solvent-rich mixtures).

The behavior of the system depends on the strengths of interactions between all single entities: polymer segments, segments of Janus dimers, solvent molecules, as well as their interactions with a solid surface.

In this work, we assume that self-interactions between fluids (oil–oil and solvent–solvent) are attractive with $\varepsilon_{WW}^{*}=1.0$, $\varepsilon_{OO}^{*}=1.5$ ($r_{cut(WW)}^{*}=r_{cut(OO)}^{*}=2.5$), while the cross-interactions are repulsive and $\varepsilon_{WO}^{*}=1$ ($r_{cut(WO)}^{*}=1.0$). Under the considered conditions (temperature and oil concentration), we observed a phase separation in the bulk oil–solvent mixtures. The oil molecules form droplets surrounded by solvent molecules.

Moreover, we assume the solvent *W* is inert with respect to the substrate ($\varepsilon_{WS}^{*}=1$, $r_{cut(WS)}^{*}=1.0$) and changes the interactions of oil (*O*) with the surface from inert ($\varepsilon_{OS}^{*}=1$, $r_{cut(OS)}^{*}=1.0)$ to more and more attractive: $\varepsilon_{OS}^{*}=0.25,\;0.5,\;0.75,\;1.0,\;1.25,\;1.5,\;1.75,$ and 2.0 ($r_{cut(OS)}^{*}=2.5$).

Firstly, we studied systems involving pure solvents and carried out simulations for different oil–surface interaction parameters, $\varepsilon_{OS}^{*}$, and the remaining parameters were fixed. The behavior of polymers on surfaces depends also on the nature of the solvent, which, in turn, depends on many parameters such as pH, salinity, the presence of active compounds, and many others. Our approach can model aqueous solutions with various additives.

However, in the second series of simulations, Janus nanoparticles were suspended in the solvent adsorbed on the inert surface. We study how the oil–Janus dimer interactions affect the detachment of oil droplets from the substrate.

### 2.2. Attachment of Oil Droplets on Solid Surfaces

We focus on the effect of oil–surface interactions on the behavior of oil droplets at the surface. The interaction of the surface with oil molecules depends on the nature of the substrate, its heterogeneity, the presence of various admixtures, active groups, etc. The location and shape of the oil cluster result from a complex competition between the interactions of polymers with the solvent and the surface. In this study, the affinity of oil to the surface is determined by the Lennard–Jones parameter $\varepsilon_{OS}^{*}$ (see Equation (2)). Our approach enables very good quantitative characterization of the adsorbed droplet and a qualitative description of changes in the contact angle.

**Figure 1:**
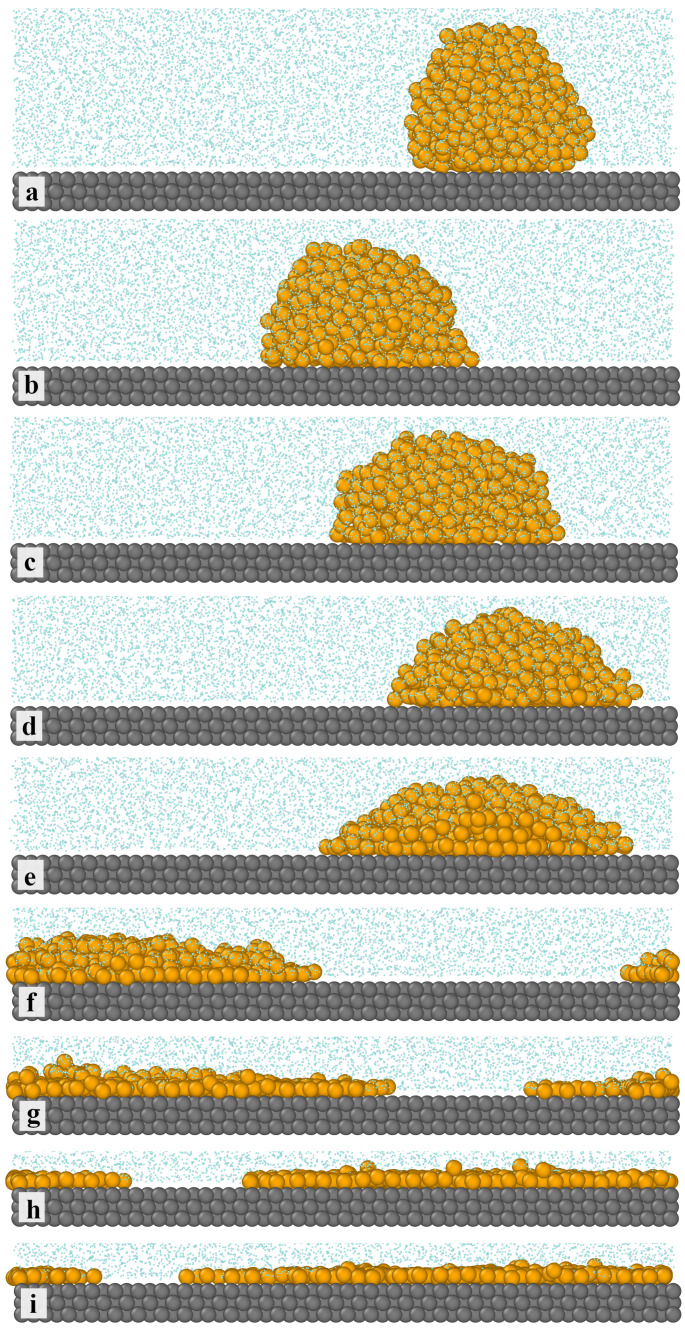
Examples of the equilibrium configurations of oil aggregates adsorbed on the surfaces of different strengths of oil–substrate interactions—(**a**) inert surface, (**b**–**i**) attractive surfaces, $\varepsilon_{OS}^{*}$: (**b**) 0.25, (**c**) 0.5, (**d**) 0.75, (**e**) 1.0, (**f**) 1.25, (**g**) 1.5, (**h**) 1.75, and (**i**) 2.0. The yellow spheres represent the polymer segments (*O*), the light blue spheres correspond to the solvent (*W*), and the gray spheres represent the solid. For greater clarity of the drawing, the solvent molecules have been reduced.

We have monitored the snapshots of configurations and determined how the oil–surface interactions affect the behavior of the system under consideration. Figure 1 shows examples of equilibrium configurations for different values of $\varepsilon_{OS}^{*}$. As the solid surface is inert with respect to oil molecules (a), the droplet is adsorbed on the surface due to hydrophobic interactions. Contacts with the surface are energetically more profitable than contacts with the surrounding solvent molecules. In this case, the droplet has the shape of a truncated and vertically elongated ball, with a contact angle of approximately $135^{\circ }$. For attractive surfaces (b–i), with the increasing strength of the OS interactions, the shape of the droplet gradually changes. The droplet becomes flatter and flatter, the oil spreads on the substrate, and islands of multilayer (f,g) and, finally, monolayer (i) films are formed on the surface. Then, the contact angle decreases to $0^{\circ }$. This reflects a change in the surface wettability, from water-wet to oil-wet.

To confirm our observations, we computed selected physical quantities characterizing these systems: the density profiles, the thickness of oil layers, and shape metrics defined by means of the gyration tensor [25].

**Figure 2:**
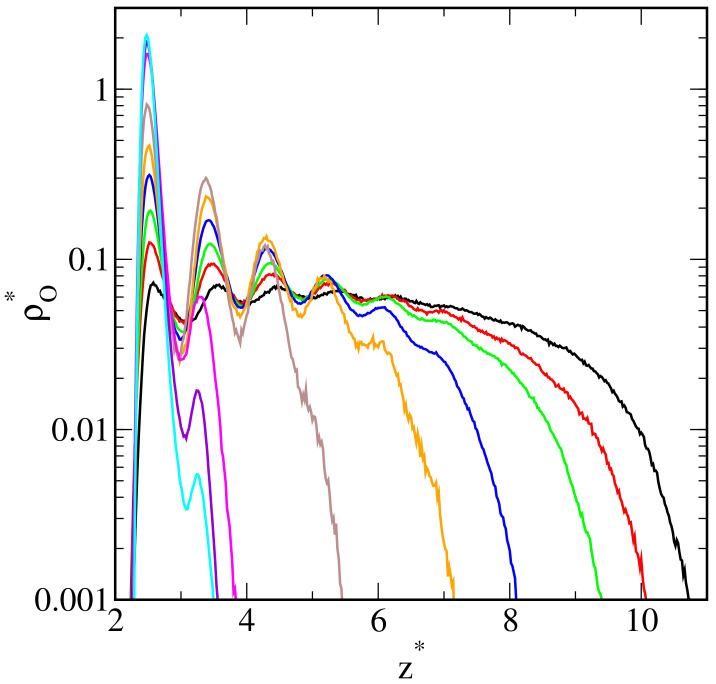
Density profiles of the polymer segments on the surfaces with different strengths of oil–substrate interactions—inert surface (black), $\varepsilon_{OS}^{*}$: 0.25 (red), 0.5 (green), 0.75 (blue), 1.0 (orange), 1.25 (brown), 1.5 (magenta), 1.75 (purple), and 2.0 (light blue). The abscissa is scaled logarithmically.

In Figure 2, we present the density profiles of polymer segments along the *z*-direction (vertical to the substrate) plotted for the systems from Figure 1. To present even very subtle changes in the density profiles, the density axis is scaled logarithmically. It is visible that an increase in the parameter $\varepsilon_{OS}^{*}$ causes a marked change in the density profiles. For the inert surface (black line), the density profile rapidly increases to an almost constant value (at $z^{*}=2.24$) and slowly decreases to zero (at $z^{*}=10.74$). Moreover, we see here five low maxima corresponding to subsequent layers of polymer segments which are visible in Figure 1. As $\varepsilon_{OS}^{*}$ changes from 0.25 to 2.0, these maxima become more pronounced and the density drops to zero closer to the surface. The profiles show a liquid-like structure of the adsorbed droplet. In the case of $\varepsilon_{OS}^{*}=1.25$, we see three peaks that indicate the existence of a three-layer structure of polymer segments (see Figure 1f). However, for the strongest surfaces, the profiles have two maxima: the first at $z^{*}=2.48$ and the much lower one at $z^{*}=3.28$. This reflects the formation of two adsorption layers. The second layer is gradually disappearing (see Figure 1i).

**Figure 3:**
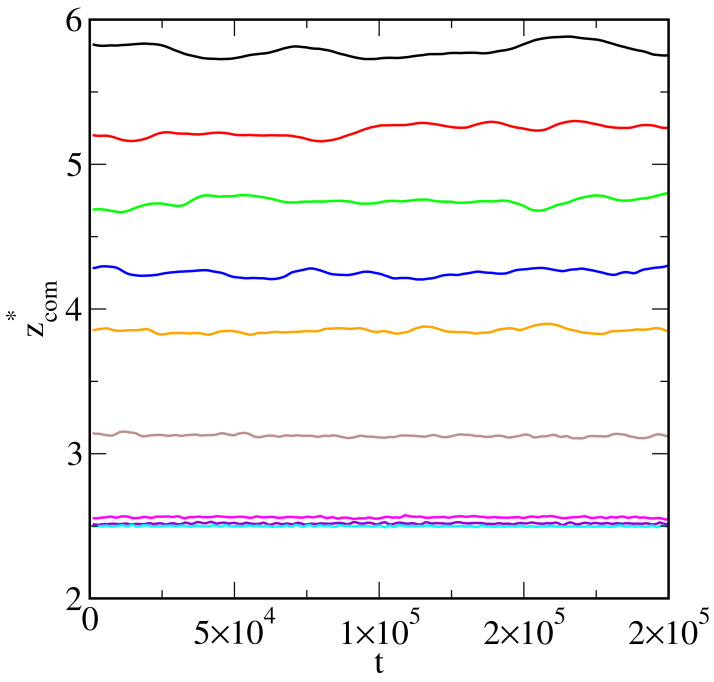
The average *z*-coordinate of the center of mass of polymer segments (*O*) as a function of time for the surfaces with different strengths of oil–substrate interactions. The results correspond to the systems from Figure 1 and Figure 2. Line colors as in Figure 2.

We confirmed that our systems achieved equilibrium by considering the changes in the average *z*-coordinate of the oil drop center of mass over time, $z_{com}^{*}$. Indeed, this parameter fluctuates around a constant value (see Figure 3). One can see that with the increasing energy of oil–surface interactions, the distance $z_{com}^{*}$ decreases. Moreover, the fluctuation amplitude becomes smaller. On the inert surface, the droplet behaves as a slightly “bouncing ball”.

**Figure 4:**
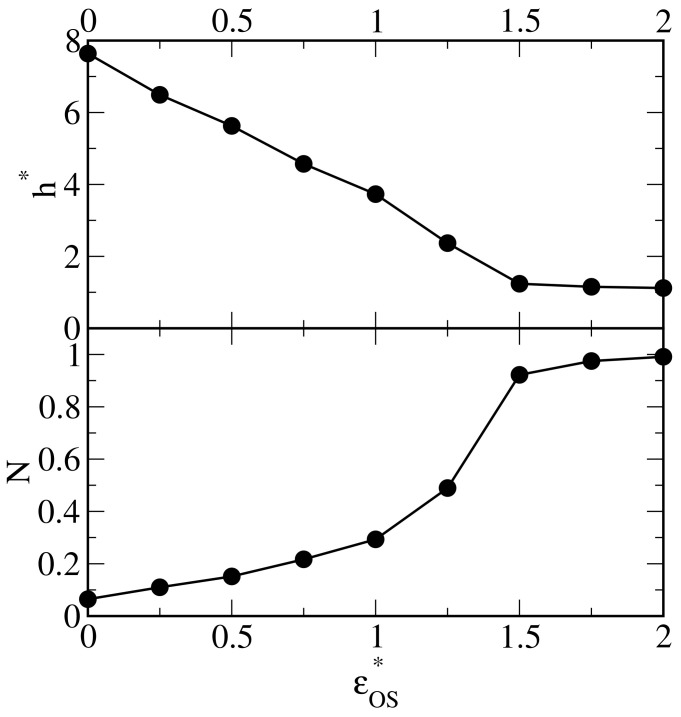
The height (thickness) of the polymer layer (**top** panel) and the number of segments in contact with the surface (*N*) (**bottom** panel) as functions of the energy parameter characterizing the oil–substrate interactions, $\varepsilon_{OS}^{*}$.

**Figure 5:**
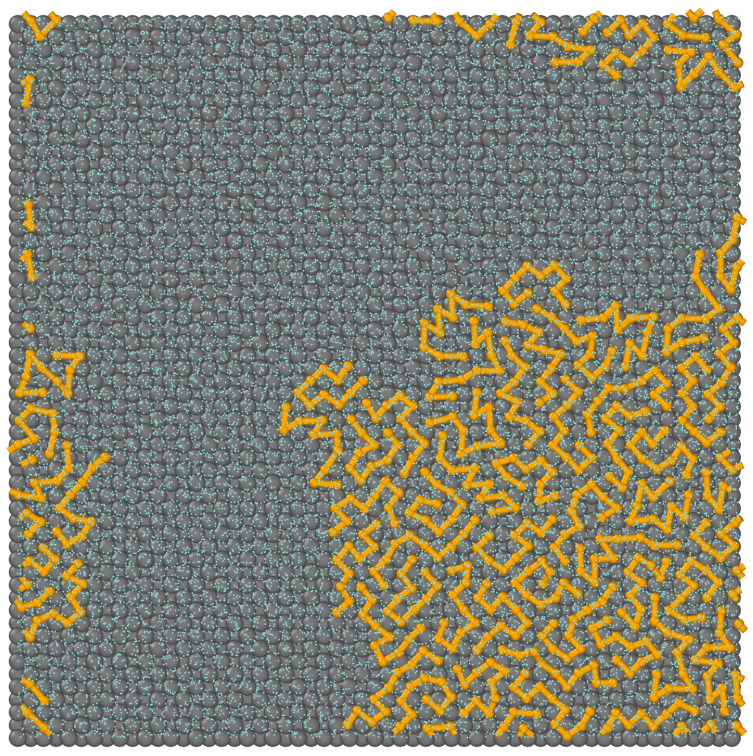
A view from above of the system (i) from Figure 1. The yellow spheres represent the polymer segments (*O*), the light blue spheres correspond to the solvent (*W*), and the gray spheres represent the solid. For greater clarity of the presentation, the solvent molecules and segments have been reduced, and the bonds between the segments have been drawn.

We have also analyzed the thickness of the polymer layer $h^{*}$ (defined by Equation (4)) and the number of segments in contact with the surface (*N*) for all discussed systems. The effect of oil–surface interactions on these characteristics is shown in Figure 4. As the energy parameter $\varepsilon_{OS}^{*}$ increases, the thickness $h^{*}$ decreases almost linearly and achieves a plateau: $h^{*}=1$ for $\varepsilon_{OS}^{*}=1.5$. Conversely, in the case of stronger adsorbents, the number of segments in close proximity to the surface increases, and eventually all segments are located on the substrate. A view from the top of the latter system is presented in Figure 5. To show the bonds, the segment diameters have been reduced. We see that the chains are lying on the surface. There are no ordered structures; the chains are chaotically distributed.

**Figure 6:**
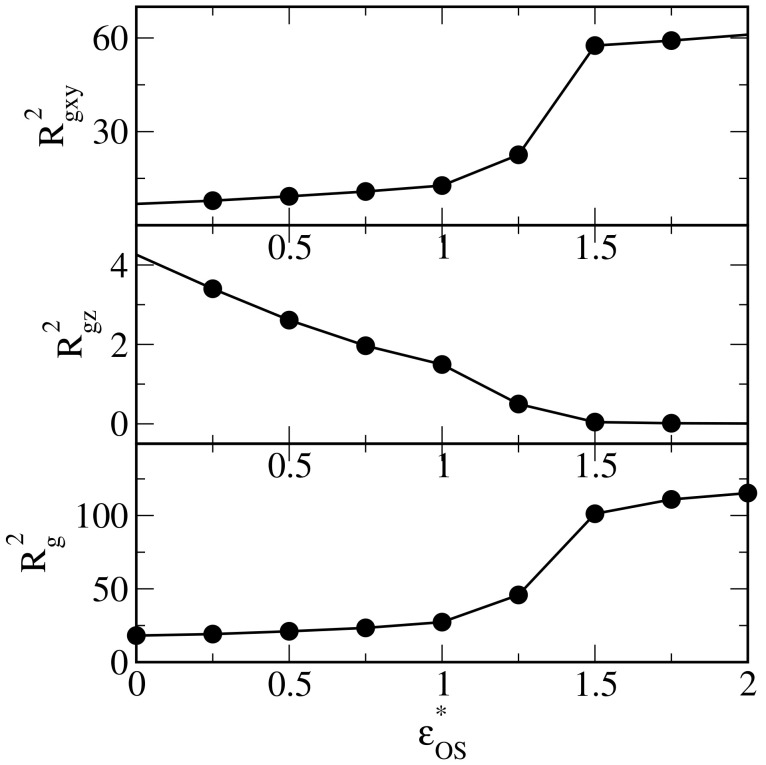
The radius of gyration in directions parallel to the surface (**top** panel), the component of the radius of gyration in the *z*-direction (**middle** panel), and the total radius of gyration (**bottom** panel) as functions of the energy parameter $\varepsilon_{OS}^{*}$.

Now, we turn out to analyze the shape of the polymer droplets adsorbed on different surfaces. We begin with the discussion of the radius of gyration calculated for clusters of adsorbed polymer segments. In Figure 6, the radius of gyration in directions parallel to the surface (top panel), the component of the radius of gyration in the *z*-direction (middle panel), and the total radius of gyration (bottom panel) are shown as functions of the energy parameter $\varepsilon_{OS}^{*}$. The radii of gyration $R_{gxy}^{*}$ and $R_{g}^{*}$ increase to certain fixed values. Conversely, the radius $R_{gz}^{*}$ decreases to a plateau for $\varepsilon_{OS}^{*}>1.5$. The oil droplets spread on stronger surfaces. Note that $R_{g}^{*}>>R_{gz}^{*}$ and $R_{gxy}^{*}>>R_{gz}^{*}$.

**Figure 7:**
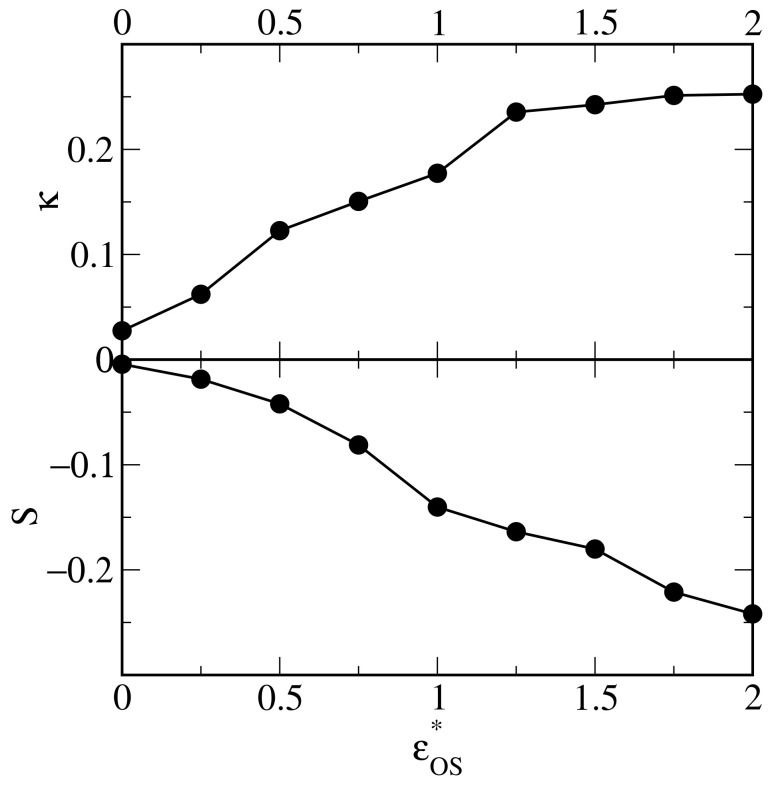
The shape anisotropy (**top** panel) and prolateness (**bottom** panel) as functions of the energy parameter $\varepsilon_{OS}^{*}$.

Other shape metrics are shown in Figure 7: the shape anisotropy (top panel) and prolateness (bottom panel). For inert surfaces, κ≈0; this means that the droplet is almost spherical. Then, the asphericity increases to 0.25 for $\varepsilon_{OS}^{*}>1.25$ indicating the existence of an almost two-dimensional cluster. We see in Figure 7 that for an inert surface, the prolateness, *S*, is close to zero and decreases gradually to −0.25. This additionally confirms the change in shape, from an almost spherical droplet to a flat aggregate.

**Figure 8:**
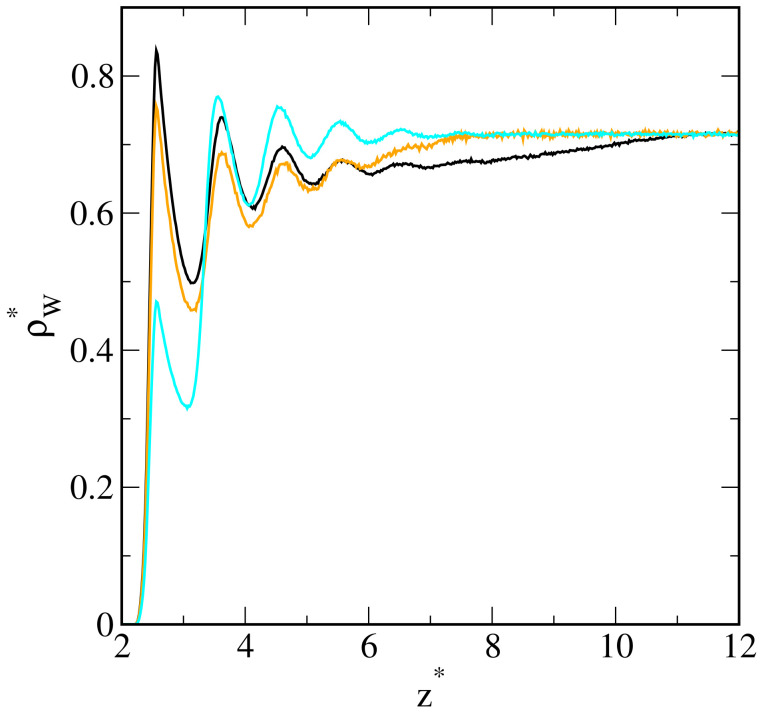
Density profiles of the solvent on the surfaces with different strengths of oil–substrate interactions—inert surface (black), $\varepsilon_{OS}^{*}$: 1.0 (orange) and 2.0 (light blue).

It is interesting to discuss the density profiles of the solvent near the surface (see Figure 8). With increasing energy $\varepsilon_{OS}^{*}$, the solvent density decreases, while the oil density increases (compare Figure 2). This quantitatively confirms the competitive adsorption of oil from the solution.

Our simulations give a molecular picture of the attachment of oil aggregates on different solid surfaces.

### 2.3. Nanoparticle-Enriched Detachment of Oil Droplets on Solid Surfaces

In this section, we report the results associated with systems involving Janus particles. We study the detachment of oil droplets from solid surfaces under static Janus nanoparticle suspension interactions. We performed simulations for the system (a) from Figure 1, to which we inserted Janus nanoparticles. Thus, we assume that the surface is inert with respect to both segments ($\varepsilon_{AS}^{*}=\varepsilon_{BS}^{*}=1.0$, $r_{cut(iS)}^{*}=\sigma_{ij}$, i=A,B). Moreover, we assume that segments A are inert with respect to water and segments B are inert with respect to oil ($\varepsilon_{AW}^{*}=\varepsilon_{BO}^{*}=1$ and $r_{cut(AW)}^{*}=r_{cut(BO)}^{*}=1.0$). In the studied systems, the concentration of Janus particles is the same: $\rho_{JP}^{*}=0.0151$.

We focus on the role of the interactions of Janus particles with the molecules of two liquids, *O* and *W*. The behavior of Janus particles at the fluid–fluid interface has been the subject of extensive studies [22,23,26]. In this context, Janus particles can be divided into two groups, taking into account the interactions of the two sides of the Janus particles with both fluids: the particles with (i) symmetrical and (ii) non-correlated interactions [26]. In the first case, the segments *A* attract apolar molecules *O* with the same strength that the segments *B* attract polar molecules *W*; $\varepsilon_{AO}^{*}=\varepsilon_{BW}^{*}$. This means that the two sides of the particle have identical deviations of apolarity and polarity from neutral wetting (the so-called supplementary wettability condition) [26]. For the second kind of particles, $\varepsilon_{AO}^{*}\neq \varepsilon_{BW}^{*}$.

In this work, we deal with the symmetrical Janus dimers and consider two values of the parameter characterizing interactions of Janus dimers with *W* and *O*: $\varepsilon_{AO}^{*}=\varepsilon_{BW}^{*}=2.0$ and 3.0.

**Figure 9:**
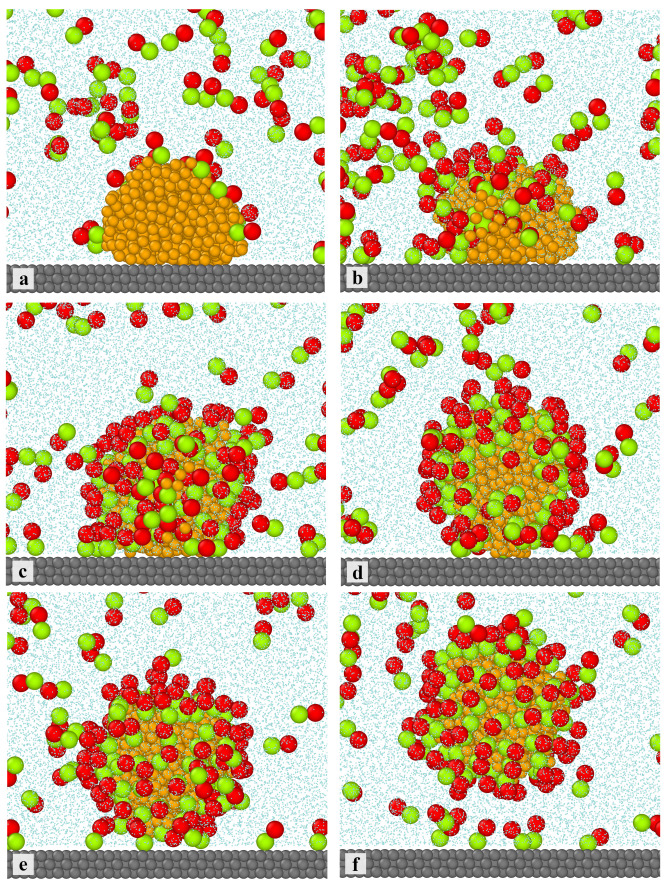
The time evolution of the system involving Janus dimers. The simulation time: 0 (**a**), $2\cdot 10^{6}$ (**b**), $8\cdot 10^{6}$ (**c**), $1.2\cdot 10^{7}$ (**d**), $1.5\cdot 10^{7}$ (**e**), and $2\cdot 10^{7}$ (**f**). The substrate is inert with respect to water and oil (compare Figure 1a). Janus dimers strongly interact with both fluids; $\varepsilon_{AO}^{*}=\varepsilon_{BW}^{*}=2.0$. The green spheres represent sebments *B*, red spheres represent sebments *A*, yellow spheres represent the polymer segments (*O*), the light blue spheres correspond to the solvent (*W*), and the gray spheres represent the solid. For greater clarity of the drawing, the solvent molecules have been reduced.

Let us consider the nanoparticle-enriched detachment of the oil droplet from the inert surface (see Figure 1a). In this case, the oil droplet is adsorbed on the surface. Figure 9 shows the time evolution of the system for Janus dimers strongly interacting with the droplet. We see that Janus particles are adsorbed on the oil droplet, with the segments *B* directed outwards. Initially, only single dimers are adsorbed on the top of the droplet. Then, Janus particles gradually cover the surface of the oil droplet exposed to the solvent. Finally, Janus particles displace oil molecules on the solid surface. They squeeze between the droplet and the surface, which leads to its deformation and a gradual detachment of oil molecules, as shown in parts d–f. The segments *B* attract solvent molecules. In this way, a kind of wedge is created which causes the droplet to detach from the surface. Moreover, the solvent molecules penetrate into the oil droplet and propagate onto the substrate. The water channels are observed in previous simulations [20]. Under appropriate conditions, droplet disruption can occur.

**Figure 10:**
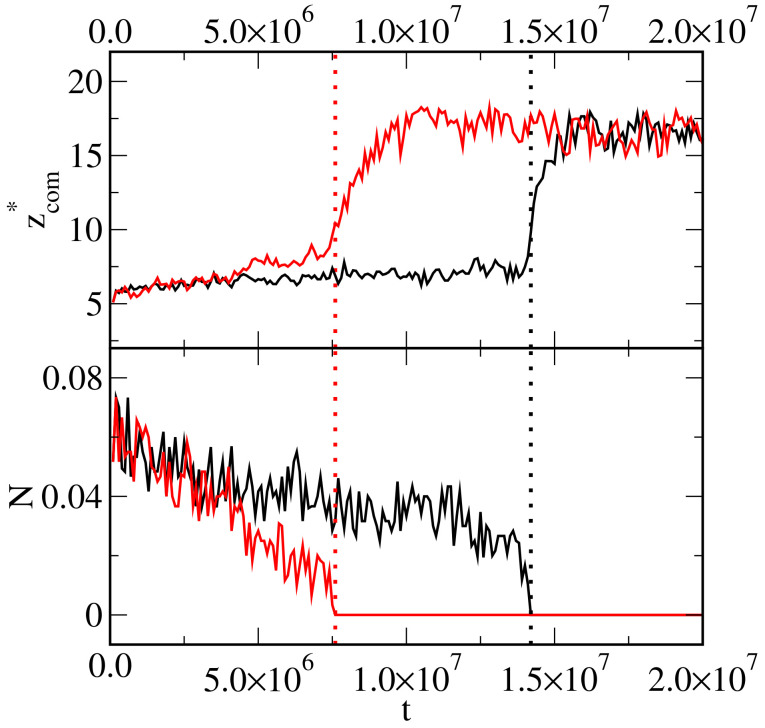
The average *z*-coordinate of the center of mass of the droplet (**top** panel) and the number of oil segments on the surface (**bottom** panel) as functions time. The substrate is inert with respect to oil, and $\varepsilon_{AO}^{*}=\varepsilon_{BW}^{*}=2.0$ (black lines) and 3.0 (red lines). The moments of detachment of the drop are marked with vertical dashed lines.

In Figure 10, we show how the average *z*-coordinate of the center of mass of the droplet (top panel) and the number of oil segments on the surface (bottom panel) change over time. We see that at any moment, $z_{com}^{*}$ increases rapidly, and the drop breaks away from the surface. Obviously, at the same time, the parameter N decreases to zero. The moment of detachment of the drop is marked with a vertical dashed line. As interactions of Janus particles with oil molecules are stronger (red lines), the droplet is removed considerably earlier than for weaker interactions (black lines).

## 3. Methods

### 3.1. Model

Therein, we consider a system containing polymers and solvent molecules in contact with a solid surface. The polymers mimic oil molecules (*O*). Under the assumed thermodynamic conditions, the oil droplets are immersed in a polar solvent (*W*). Moreover, we study systems with Janus particles (JP) suspended in the solvent. A simple coarse-grained model is used to represent all species.

We assume that polymer segments and solvent molecules have the same diameters, σ. All polymer chains consist of *M* segments. The chain connectivity is assured by imposing a finitely extensible nonlinear elastic (FENE) segment–segment potential

(1) $$U_{FENE}=-\frac{k}{2}R_{0}^{2}ln\left[1-{\left(\frac{r}{R_{0}}\right)}^{2}\right]$$

where *r* denotes the separation distance between the segments, *k* is the spring constant, and $R_{0}$ is the maximum possible length of the spring. The standard parameters of the binding potential (1) are assumed to be k=30 and $R_{0}=1.5$.

The Janus particle is a dimer composed of two spheres (segments) *A* and *B*. These spheres have the same diameters ($\sigma_{JP}$) [22,23,26]. The solid is modeled as “atoms” (*S*) of diameters $\sigma_{S}=\sigma$, arranged on an fcc lattice. The crystalline plane (0 0 1) is considered an exposed surface.

The interactions between all “atoms” (solvent molecules, chain segments, and solid molecules) are described via the shifted-force Lennard–Jones potential [24]

(2) $$u=\left\{\begin{array}{cc} 4\varepsilon_{ij}\left[{\left(\sigma_{ij}/r\right)}^{12}-{\left(\sigma_{ij}/r\right)}^{6}\;\right]-\Delta u\left(r\right), & \;\;\;r<r_{cut(ij)},\\ 0, & \;\;\;\mathrm{otherwise}, \end{array}\right.$$

where

(3) $$\Delta u\left(r\right)=u\left(r_{cut(ij)}\right)+\left(r-r_{cut(ij)}\right)\partial u\left(r_{cut(ij)}\right)/\partial r,$$

and $r_{cut(ij)}$ is the cutoff distance, $\sigma_{ij}=0.5\left(\sigma_{i}+\sigma_{j}\right)$, (i,j=O,W,A,B,S), and $\varepsilon_{ij}$ denotes the parameter that characterizes the interaction strengths between spherical species *i* and *j*. The indices *O*, *W*, *A*, *B*, and *S* correspond to oil, a polar fluid, the segments of Janus dimer, and the solid, respectively. We use the cutoff distance to switch on or switch off attractive interactions. For attractive interactions, $r_{cut(ij)}=2.5\sigma_{ij}$, while for repulsive interactions, $r_{cut(ij)}=\sigma_{ij}$.

### 3.2. Definitions and Formulas Used

We introduce the standard units commonly used in coarse-grained simulations. The diameter of solvent atoms is the distance unit, σ, the mass of a solvent atom is the mass unit, $m_{W}=m$, while the solvent–solvent energy parameter, $\varepsilon_{WW}$, is the energy unit. The basic unit of time is $\tau =\sigma {\left(\varepsilon /m\right)}^{1/2}$. The gravity effect is assumed to be negligible.

Moreover, we use the reduced quantities, reduced distances $l^{*}=l/\sigma$, and reduced energies $E^{*}=E/\varepsilon$, and the reduced temperature is $T^{*}=k_{B}T/\varepsilon$, where $k_{B}$ is the Boltzmann constant.

We define also the reduced density of the kth component as $\rho_{k}^{*}=\rho_{k}\sigma_{k}^{3}$, where $\rho =N_{k}/V$ is its number density, $N_{k}$ is a total number of “atoms” *k*, and *V* is the volume of the system. During simulations, we have estimated the reduced density profiles of all species.

To characterize the behavior of oil chains adsorbed on the surface, we also calculated the average *z*-coordinate of the center of mass of oil segments ($z_{com}$), the number of oil segments which “touch” the surface, that is, those that are located at a distance from the surface smaller than 0.7 (*N*), the average height (thickness) of the adsorbed layer (*h*), and the selected shape metrics of the oil aggregate (droplet) [25].

It has been a widespread practice to define the height of the adsorbed polymer layer, *h*, from the first moment of the total segment density profile [27]

(4) $$h=2\frac{\int dzz\rho_{O}\left(z\right)}{\int dz\rho_{O}\left(z\right)}$$

We use the shape characteristics defined by means of the gyration tensor [25]

(5) $$G_{\alpha \beta }=\frac{1}{N^{\prime }}\langle \sum_{i=1}^{N^{\prime }}\left(r_{i,\alpha }-r_{0,\alpha }\right)\left(r_{i,\beta }-r_{0,\beta }\right)\rangle$$

where $r_{i,\alpha }$ and $r_{0,\alpha }$ are the α component (α,β=x,y,z) of the ith segment and the center of mass of the segment aggregate (droplet), respectively.

Diagonalization of the gyration tensor yields its eigenvalues $\lambda_{i}$ (i=1,2,3), which we order as $\lambda_{1}\geq \lambda_{2}\geq \lambda_{3}$. Then, three invariants can be obtained: $I_{1}=\lambda_{1}+\lambda_{2}+\lambda_{3}$, $I_{2}=\lambda_{1}\lambda_{2}+\lambda_{1}\lambda_{3}+\lambda_{2}\lambda_{3}$, and $I_{3}=\lambda_{1}\lambda_{3}\lambda_{3}$. We use these values to define the shape descriptors of the segment aggregate [25].

The radius of gyration of the aggregate of the segments is given as

(6) $$R_{g}^{2}=I_{1}=\frac{1}{N^{\prime }}\langle \sum_{i=1}^{N^{\prime }}r_{i0}^{2}\rangle$$

where $r_{i0}=r_{i}-r_{0}$, and $r_{i}$ and $r_{0}$ are positions of the ith segment and the center of mass, respectively, and $N^{\prime }$ is the number of segments in the cluster.

We resolved the vectors $r_{i0}$ in Equation (6) into components parallel to the axes *x*, *y*, and *z*, and calculated the corresponding radii of gyration labeled $R_{g\alpha }^{2}$ (α=x,y,z), the sum of which is equal to $R_{g}^{2}$. Moreover, we computed the radius of gyration in directions parallel to the solid surface, $R_{gxy}^{2}=R_{gx}^{2}+R_{gy}^{2}$.

The relative shape anisotropy is defined as

(7) $$\kappa =1-3\langle I_{2}/I_{1}^{2}\rangle .$$

The relative shape anisotropy takes values between 0 (perfectly spherical objects) and 1 (rigid rods). However, for a regular planar array, κ=0.25 [25].

The prolateness is given by

(8) $$S=\langle \left(3\lambda_{1}-I_{1}\right)\left(3\lambda_{2}-I_{1}\right)\left(3\lambda_{3}-I_{1}\right)/I_{1}^{3}\rangle$$

with its value varying from 0.25 to 2, where the negative values indicate oblate shapes, whereas the positive ones correspond to prolate objects [25].

### 3.3. Simulation Method

In this work, all molecular dynamics (MD) simulations were carried out using the LAMMPS package [28,29]. The reduced temperature was maintained at $T^{*}=1$ using the Nosé–Hoover thermostat. The time step was set to 0.002.

Simulations were carried out in a rectangular box of reduced dimensions equal to $L_{x}^{*}=L_{x}^{*}=40$ and $L_{x}^{*}=28$ along the axes *x*, *y*, and *z*, respectively. Standard periodic boundary conditions in the *x* and *y* directions were assumed. The box was closed by a reflective wall. The distance $L_{z}^{*}$ was large enough not to influence the behavior of the drop. The solid was located at the bottom of the box. We placed here 4873 “atoms” *S* to form an fcc lattice. We considered an ensemble of 28,000 solvent molecules (*W*) and 60 decan molecules (M=10) that mimic oil (*O*). The reduced density of the solvent was $\rho_{W}^{*}=0.7$.

Firstly, we performed simulations for the bulk system (without interactions with the solid surface). Under the assumed conditions, we observed the formation of the oil droplet. This configuration was used as the initial configuration in the simulation of the behavior of the oil droplet on different surfaces.

In the second series of simulations, we added $N_{JP}=100$ Janus dimers to the system with the oil droplet attached to the surface and observed its time evolution. The reduced diameters of the segments of Janus dimers were set $\sigma_{A}^{*}=\sigma_{B}^{*}=1.5$.

The simulated systems comprised 33,473 or 33,673 “atomic units”. We equilibrated each system for at least $10^{7}$ time steps until its total energy reached a constant level, at which it fluctuated around a mean value. The production runs were for at least $10^{6}$ time steps.

The visualization was implemented by the OVITO 3.0.0 software [30].

## 4. Conclusions

Adopting the molecular dynamic simulation method, the deposition of oil droplets on the solid surfaces and the detachment performance were investigated. Simulations show how oil–substrate interactions affect the behavior of oil aggregates on different surfaces. These interactions control the wettability of the surface. Simulations demonstrate that the surface wettability can change from water-wet to oil-wet. Using selected characteristics, the structure of the adsorbed aggregates was analyzed. For oil clusters, we calculated the radius of gyration and its components, asphericity, prolateness, and the average thickness of the adsorbed layer.

In the case of the solid surface that is inert with respect to oil molecules, the droplet resembles a truncated sphere. For attractive surfaces, however, with the increasing strength of the OS interactions, the droplet becomes flatter and flatter, the oil spreads on the substrate, and islands of multilayer and, finally, monolayer films are observed on the surface. The density profiles of the oil and solvent reflect these changes and confirm a competitive character of adsorption in oil–aqueous solvent mixtures. On the surface of a strong adsorbent, polymers that mimic oil molecules are lying on the surface.

Moreover, we studied the nanoparticle-enriched detachment of the oil droplet from the surface. Janus dimers (AB) with supplementary wettability of segments [22] were used as the active agent. We discussed the time evolution of the system after inserting these particles. The Janus dimers were adsorbed on the oil droplet with the segments *B* directed outwards and they gradually covered the surface of the oil droplet exposed to the solvent. Then, the Janus particles displaced the oil molecules on the solid surface. The segments *B* attracted the solvent molecules that also replaced the oil molecules on the surface and penetrated the oil droplet. As a result, the droplet was detached from the surface. As the interactions of the Janus particles with the oil molecules were stronger, the detachment process was faster.

The proposed method can be used to study oil detachment from strongly attractive surfaces by various active agents like surfactants, Janus nanoparticles, and hairy nanoparticles. Such systems are currently under study in our laboratory.

Our simulations provide a picture of the attachment and detachment of oil droplets on different solid surfaces at the molecular level. The discussion of the results gives an in-depth understanding of the underlying mechanisms of these processes.

## Data Availability

The raw data supporting the conclusions of this article will be made available by the authors on request.

## References

[1] Ratanpara A., Kim M. (2023). Wettability Alteration Mechanisms in Enhanced Oil Recovery with Surfactants and Nanofluids: A Review with Microfluidic Applications. Energies.

[2] Wu Y.S. (2016). Chapter 2—Multiphase Fluids in Porous Media. Multiphase Fluid Flow in Porous and Fractured Reservoirs.

[3] Rackley S.A. (2017). 18—Other geological storage options. Carbon Capture and Storage.

[4] Perera M.S.A., Gamage R.P., Rathnaweera T.D., Ranathunga A.S., Koay A., Choi X. (2016). A Review of CO_2_-Enhanced Oil Recovery with a Simulated Sensitivity Analysis. Energies.

[5] Sun X., Zhang Y., Chen G., Gai Z. (2017). Application of Nanoparticles in Enhanced Oil Recovery: A Critical Review of Recent Progress. Energies.

[6] Sanni M. (2018). Petroleum Engineering: Principles, Calculations and Workflows.

[7] Mohammed M., Babadagli T. (2015). Wettability alteration: A comprehensive review of materials/methods and testing the selected ones on heavy-oil containing oil-wet systems. Adv. Colloid Interface Sci..

[8] Kamal M.S., Hussein I.A., Sultan A.S. (2017). Review on Surfactant Flooding: Phase Behavior, Retention, IFT, and Field Applications. Energy Fuels.

[9] Eltoum H., Yang Y.L., Hou J.R. (2021). The effect of nanoparticles on reservoir wettability alteration: A critical review. Pet. Sci..

[10] Chowdhury S., Shrivastava S., Kakati A., Sangwai J.S. (2022). Comprehensive Review on the Role of Surfactants in the Chemical Enhanced Oil Recovery Process. Ind. Eng. Chem. Res..

[11] Zhang M., Nan Y., Lu Y., You Q., Jin Z. (2023). CO_2_-responsive surfactant for oil-in-water emulsification and demulsification from molecular perspectives. Fuel.

[12] Lim S., Horiuchi H., Nikolov A.D., Wasan D. (2015). Nanofluids Alter the Surface Wettability of Solids. Langmuir.

[13] Li S., Torsæter O., Lau H.C., Hadia N.J., Stubbs L.P. (2019). The Impact of Nanoparticle Adsorption on Transport and Wettability Alteration in Water-Wet Berea Sandstone: An Experimental Study. Front. Phys..

[14] Lei W., Lu X., Wang M. (2023). Multiphase displacement manipulated by micro/nanoparticle suspensions in porous media via microfluidic experiments: From interface science to multiphase flow patterns. Adv. Colloid Interface Sci..

[15] Cheraghian G., Hendraningrat L. (2016). A review on applications of nanotechnology in the enhanced oil recovery part A: Effects of nanoparticles on interfacial tension. Int. Nano Lett..

[16] Fu L., Gu F., Liao K., Wen X., Jiang L., Li X., Huang W., Shao M. (2022). Molecular dynamics simulation of enhancing surfactant flooding performance by using SiO_2_ nanoparticles. J. Mol. Liq..

[17] Vu T.V., Papavassiliou D.V. (2019). Synergistic effects of surfactants and heterogeneous nanoparticles at oil-water interface: Insights from computations. J. Colloid Interface Sci..

[18] Liang S., Fang T., Xiong W., Ding B., Yan Y., Zhang J. (2019). Oil detachment by modified nanoparticles: A molecular dynamics simulation study. Comput. Mater. Sci..

[19] Chang Y., Xiao S., Ma R., Wang X., Zhang Z., He J. (2022). Displacement dynamics of trapped oil in rough channels driven by nanofluids. Fuel.

[20] Zhou W., Tang X., Liu X., Yan Y., Chen C. (2024). Interfacial Behaviors and Oil Detachment Mechanisms by Modified Silica Nanoparticles: Insights from Molecular Simulations. J. Phys. Chem. B.

[21] Tohidi Z., Teimouri A., Jafari A., Gharibshahi R., Omidkhah M.R. (2022). Application of Janus nanoparticles in enhanced oil recovery processes: Current status and future opportunities. J. Pet. Sci. Eng..

[22] Kang C., Honciuc A. (2018). Influence of Geometries on the Assembly of Snowman-Shaped Janus Nanoparticles. ACS Nano.

[23] Borówko M., Słyk E., Sokołowski S., Staszewski T. (2019). Janus Dimers at Liquid–Liquid Interfaces. J. Phys. Chem. B.

[24] Toxvaerd S., Dyre J.C. (2011). Communication: Shifted forces in molecular dynamics. J. Chem. Phys..

[25] Theodorou D.N., Suter U.W. (1985). Shape of unperturbed linear polymers: Polypropylene. Macromolecules.

[26] Park B.J., Lee D. (2012). Equilibrium Orientation of Nonspherical Janus Particles at Fluid–Fluid Interfaces. ACS Nano.

[27] Pastorino C., Binder K., Müller M. (2009). Coarse-Grained Description of a Brush-Melt Interface in Equilibrium and under Flow. Macromolecules.

[28] Plimpton S. (1995). Fast Parallel Algorithms for Short-Range Molecular Dynamics. J. Comput. Phys..

[29] Thompson A.P., Aktulga H.M., Berger R., Bolintineanu D.S., Brown W.M., Crozier P.S., In ’t Veld P.J., Kohlmeyer A., Moore S.G., Nguyen T.D. (2022). LAMMPS—A flexible simulation tool for particle-based materials modeling at the atomic, meso, and continuum scales. Comp. Phys. Comm..

[30] Stukowski A. (2010). Visualization and analysis of atomistic simulation data with OVITO-the Open Visualization Tool. Model. Simul. Mater. Sci. Eng..

